# Temporal changes in functional outcome and case-fatality after ischaemic stroke and intracerebral haemorrhage in Sweden 2010–2019: *an observational study from the Swedish Stroke Register (Riksstroke)*

**DOI:** 10.1093/esj/aakaf021

**Published:** 2026-01-01

**Authors:** Conrad Drescher, Fredrik Buchwald, Teresa Ullberg, Mats Pihlsgård, Bo Norrving, Jesper Petersson

**Affiliations:** Neurology, Department of Clinical Sciences Lund, Lund University, and Neurology, Skåne University Hospital Lund/Malmö, Lund, Sweden; Neurology, Department of Clinical Sciences Lund, Lund University, and Neurology, Skåne University Hospital Lund/Malmö, Lund, Sweden; Neurology, Department of Clinical Sciences Lund, Lund University, and Neurology, Skåne University Hospital Lund/Malmö, Lund, Sweden; Perinatal and Cardiovascular Epidemiology, Department of Clinical Sciences Malmö, Lund University, Malmö, Sweden; Neurology, Department of Clinical Sciences Lund, Lund University, and Neurology, Skåne University Hospital Lund/Malmö, Lund, Sweden; Neurology, Department of Clinical Sciences Lund, Lund University, and Neurology, Skåne University Hospital Lund/Malmö, Lund, Sweden

**Keywords:** ischaemic stroke, ICH, functional outcome, case-fatality, temporal changes, Sweden, stroke registry

## Abstract

**Introduction:**

There are few recent studies on trends over time in functional outcome and mortality after stroke, with results separately presented for ischaemic stroke (IS) or ICH. We aimed to determine temporal changes in functional outcome and case-fatality 90 days after IS and ICH in Sweden between 2010 and 2019.

**Patients and methods:**

We included patients (≥18 years) with first-ever IS or ICH registered in the Swedish Stroke Register (Riksstroke) between 2010 and 2019. Functional outcome data were based on the Riksstroke 90-day follow-up surveys and reported as distribution on the mRS. Multiple imputation was used for missing functional status in the survey non-responders (15.2% of total cohort). Mortality data were obtained from the Swedish Cause of Death Register, and “all-cause” mortality within 90 days was used as the outcome. Logistic regression was applied to calculate odds ratios for good functional outcome (mRS 0–2), and Cox regression was used to estimate hazard ratios for death within 90 days, with 2010–2012 as the reference period. Analyses were stratified by age groups (18–64, 65–74, 75–84, ≥ 85 years) and by 3 time periods (2010–2012, 2013–2016, 2017–2019).

**Results:**

Between 2010 and 2019, 153,865 (87.3%) cases of IS and 22,289 (12.7%) cases of ICH were registered in Riksstroke. Good functional outcome (mRS 0–2) after 90 days increased in patients with IS from 49.2% in 2010–2012 to 52.4% in 2017–2019 (adjusted odds ratio [aOR] 1.12; 95% CI, 1.09–1.16) but not in patients with ICH (from 34.2% to 34.3%, aOR 0.96; 95% CI, 0.88–1.06). A significant improvement in functional outcome after IS from 2010–2012 to 2017–2019 was only observed in patients over 75 years. Crude 90-day case-fatality decreased in both IS (from 13.8% to 12.4%) and ICH (from 31.0% to 30.4%) from 2010–2012 to 2017–2019. Adjusted hazard ratios for case-fatality showed no significant changes over time for IS (0.99; 95% CI, 0.95–1.02) or ICH (1.00; 95% CI, 0.94–1.06).

**Conclusion:**

We observed improvements in functional outcome after IS but not after ICH in Sweden between 2010 and 2019. Changes over time in functional outcome were more favourable in patients older than 75 years in both IS and ICH. Case-fatality decreased in IS and ICH, but this reduction was not significant after adjustment for confounding.

## Graphical Abstract

**Graphical abstract:**
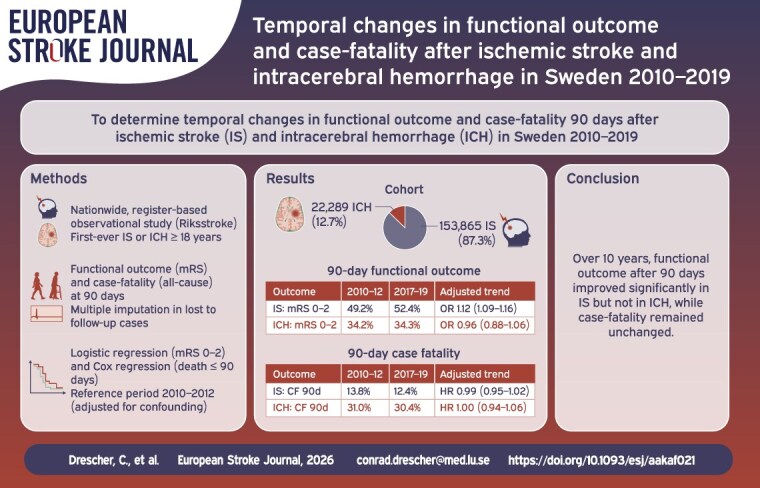

## Introduction

Stroke remains the second leading cause of death, and the third leading cause of death and disability combined among non-communicable disorders worldwide.^1^ In previous decades, the age-standardised incidence of ischaemic stroke (IS) has decreased, whereas the incidence of ICH has remained unchanged in high-income countries.^2–4^ In Sweden, the incidence of first-ever IS decreased by 17% and of first-ever ICH by 10% between 2010 and 2019.^5^^,^^6^

Functional outcomes after stroke have been investigated in several studies, but evidence on changes over time remains limited, particularly when IS and ICH are analysed separately. For IS, some previous studies demonstrated positive trends over time in functional outcome,^7–10^ whereas a recent systematic review and meta-analysis on ICH showed no improvement in functional outcome over time.^4^

Previous studies investigating changes over time in case-fatality (CF) have demonstrated a decline in CF after IS.^2^^,^^7^^,^^11^^,^^12^ However, more recent analyses suggest that the decline, which was most evident before 2010, may have slowed or even plateaued in the subsequent years.^13^ For ICH, no changes in CF over time were reported in a recent review.^4^

These temporal patterns may reflect several factors. For IS, advances in acute treatment (eg, intravenous thrombolysis, mechanical thrombectomy), improved primary and secondary preventions (eg, direct oral anticoagulants for atrial fibrillation [AF], wider use of statins and antihypertensive therapy), and better organised stroke care have likely contributed to improved outcomes and declining CF. In contrast, for ICH, fewer advances in effective acute therapies and prevention strategies may help explain the lack of improvement over time. The relative importance of these factors remains uncertain and warrants further investigation.

The aim of this register-based, nationwide study was to analyse temporal changes in functional outcome and CF after 90 days for IS and ICH in Sweden between 2010 and 2019.

## Patients and methods

### Study population

Data were obtained from the Swedish Stroke Register (Riksstroke), the Swedish quality register for stroke care. Riksstroke is a hospital-based stroke register, and all 72 Swedish acute care hospitals contribute to collecting and entering data. Between 2010 and 2019, the estimated coverage rate of Riksstroke was stable at about 90% of admitted acute stroke cases in Sweden, with no obvious differences in coverage between age groups or sexes.^14^^,^^15^

We included patients aged ≥ 18 years, hospitalised between 1 January 2010 and 31 December 2019, with an *International Classification of Diseases, Tenth Revision (ICD-10)* diagnosis of either ICH (I61) or IS (I63), and no record of a previous stroke (either ischaemic or haemorrhagic). Information about previous stroke was based on patient interview and/or available medical chart information entered into Riksstroke. We selected the period 2010–2019 based on data availability and to ensure comparability with our previous nationwide analyses of IS and ICH epidemiology using the same dataset and study period,^5^^,^^6^ allowing a coherent continuation by examining functional outcomes and CF in the same population. Patients with a diagnosis of I64 (stroke, not specified as haemorrhage or infarction) were excluded (about 1.2% of cases in Riksstroke between 2010 and 2019, patient characteristics shown in Table S2).

### Patient characteristics

Data on patient characteristics were collected during hospital stay and included sex, age, previous TIA/amaurosis fugax, cardiovascular risk factors (hypertension, diabetes mellitus, smoking and AF), medication at admission (blood pressure lowering drugs, antiplatelet drugs, anticoagulants and lipid-lowering drugs), pre-stroke functional status, stroke severity and acute reperfusion therapies (intravenous thrombolysis [IVT] and/or mechanical thrombectomy [MT]). Pre-stroke independency in activities of daily living (ADL) was defined as living in own home without home care services and being independent in dressing, toileting and indoor mobility. Stroke severity was determined by the patients’ level of consciousness at admission using the Reaction Level Scale RLS-85 with categories of alert (RLS 1), drowsy (RLS 2–3) and comatose (RLS 4–8).^16^ Data on NIHSS were missing in about 50% of patients and therefore not used in the analysis.

### Functional outcome and case-fatality after 90 days

Information on functional outcome after 90 days was obtained from Riksstroke’s 3-month follow-up survey which was distributed by mail (with 2 postal reminders to non-respondents), telephone or during a follow-up visit. The survey could be either completed by the patient alone, with caregiver assistance, or by caregiver alone. The survey collects information about dressing (independent or assistance required), toileting (independent or assistance required), mobility (fully mobile both indoors and outdoors, mobile but only indoors or fully dependent on assistance for mobility), living conditions (living independently, living independently with homecare, residing at an assisted living facility or in need of in-patient care) and dependency on next of kin for support (fully dependent on next of kin, partially dependent, not dependent or unknown dependency). We used this information to estimate mRS scores for each patient using a validated translation algorithm.^17^ The mRS is a widely used scale to evaluate functional outcome after stroke and ranges from 0 to 6, with 0 indicating no symptoms and 6 indicating death. The translation algorithm did not permit separation of mRS grades 0, 1 and 2 so we used mRS 0–2 for analysis on “good” functional outcome. Scores for mRS 3, 4 and 5 were available and could be used separately for analysis on “poor” functional outcome. Individuals not returning the questionnaire or with incomplete information on any of the variables needed for estimating the mRS score were defined as lost to follow-up.

Information on death within 90 days was obtained from the Swedish Causes of Death Register which has virtually complete coverage.^18^ Data from this register were merged with Riksstroke’s individual patient data by a statistician at the Riksstroke secretariat. Case-fatality was defined as all-cause mortality within 90 days after the index stroke event, as information on cause-specific mortality was not available. Deceased individuals were assigned as followed up with mRS 6.^19^

To look at changes in the analysed variables over time, data are presented for patients with IS and ICH for 3 different time periods (2010–2012, 2013–2016 and 2017–2019). Time periods including several years were chosen to reduce the effect of annual fluctuations and for a comprehensive presentation. Data were analysed and presented for different age groups (18–64 years, 65–74 years, 75–84 years and ≥ 85 years). Functional outcome data for IS and ICH combined are available in the Supplementary material.

### Statistical methods

Statistical analyses were performed using IBM SPSS Statistics version 28 and SAS. Categorical variables were presented as frequencies with percentage, and continuous data as medians with interquartile ranges. To analyse differences between groups, chi-square test was used for categorical variables.

Multiple imputation was performed to estimate mRS scores for patients lost to follow-up at 90 days. The imputation model included selected baseline characteristics as predictor values: sex, age, diagnosis, living conditions before stroke, previous stroke, smoking, previous transient ischaemic attack/amaurosis fugax, AF, diabetes mellitus, hypertension, level of consciousness at admission and functional status before stroke. Functional ability in toileting, dressing, mobility, as well as living conditions and need of support from next of kin, were used both as predictors and variables to be imputed. Because the data displayed a nonmonotone pattern of missing values and the missing variables were categorical, we imputed using the fully conditional specification method based on a logistic regression model.^20^ Five imputations were conducted, each based on 10 iterations of the underlying Markov chain. mRS scores were then calculated for each data set using the translation algorithm previously described, and an average was presented (Table S4). The decision to impute missing outcome data was based on the availability of extensive baseline and follow-up variables strongly associated with functional status, supporting the assumption that data were missing at random (MAR). Multiple imputation was preferred over complete-case analysis to reduce potential bias from selective loss to follow-up and to increase statistical precision, while acknowledging that imputing outcomes may introduce uncertainty if the MAR assumption is violated. To evaluate this, we performed attrition analyses comparing baseline characteristics of patients lost to follow-up with those followed up or deceased at 90 days (Table S3a) and examined changes over time in baseline characteristics among those lost to follow-up (Table S3b). We also conducted a sensitivity analysis restricted to complete cases (Table S7).

Logistic regression analysis was used to estimate odds ratios (ORs) for trends in good functional outcome (mRS 0–2) at 90 days among stroke survivors, with 2010–2012 as reference period, including imputed data. Results were presented as crude and adjusted for age, sex, stroke severity and pre-stroke independency.

Cox proportional hazards regression models were applied to estimate hazard ratios (HRs) for death within 90 days for the time periods 2013–2016 and 2017–2019 compared to 2010–2012 as reference. Follow-up time was defined from the date of stroke onset until death or censoring at day 90, with patients alive at 90 days censored on that date. The proportional hazards assumption was evaluated using the Schoenfeld residuals. Some deviations from proportionality were observed, particularly when assessing the entire cohort, but these were minor and not considered to materially influence the estimated associations. Results were presented as crude and adjusted for age, sex, stroke severity and pre-stroke independency.

Statistical significance was defined as a 2-sided *P*-value < .05, corresponding to a type I error rate of 5%.

## Results

Between 2010 and 2019, 230,577 cases of IS or ICH were registered in Riksstroke. Of these, 176,154 (76.4%) patients had no history of a previous stroke. Of these first-time events, 153,865 (87.3%) patients had IS and 22,289 (12.7%) had ICH. After 90 days, 27,428 (15.6%) patients had died, 26,802 (15.2%) were alive but lost to follow up or had incomplete outcome data and 121,919 (69.2%) were alive and followed up with complete outcome data (Figure S1). In patients followed up, 88% answered the Riksstroke’s 3-month follow-up survey alone or with the assistance of a caregiver.

### Temporal changes in patient characteristics

Changes over time in patient characteristics between 2010 and 2019 for first-ever IS and ICH are shown in Table 1. Details about trends in baseline characteristics such as age, sex and vascular risk factors for IS and ICH have been presented in previous publications.^5^^,^^6^ Data for IS and ICH when combined are shown in Table S1.

**Table 1:** Patient characteristics for first-ever ischaemic stroke and first-ever ICH for 3 different time periods

	**Ischaemic stroke**			**Intracerebral haemorrhage**		
	**2010–2012**	**2013–2016**	**2017–2019**	**2010–2012**	**2013–2016**	**2017–2019**
	*n* = 48,969	*n* = 61,508	*n* = 43,388	*n* = 6,704	*n* = 9,127	*n* = 6,458
Cases per year *n*	16,323	15,377	14,463	2,235	2,282	2,153
% of all stroke cases	88	87.1	87	12	12.9	13
Male sex (%)	50.8	51.5	52.9	52.9	53.4	55.3
Age (years) as median (IQR)	77 (67–85)	76 (68–85)	76 (68–84)	75 (64–83)	75 (65–83)	75 (65–83)
**Risk factors (%)**						
Hypertension	57.7	59.7	60.8	49	52.5	54.7
Diabetes	19.4	20.4	22.2	14.5	15.5	16.8
Smoking	14.2	14	13.2	10	9.8	9.3
AF total	27.4	27.9	27.6	19.1	22.3	24.4
Previous TIA	7.3	7.3	7.6	5	5.3	6.5
**Stroke severity (%)**						
Alert	86	86.7	87.3	59.6	61.9	61.5
Drowsy	10.1	9.3	8.9	21.8	20.5	21.2
Unconscious	2.8	2.8	2.4	17	16	15.6
**Premorbid status (%)**						
Institutional Living	6.8	7.2	6.5	7.8	7.7	7.8
ADL-independency	76.6	76.6	77.6	76.5	75.6	74.8
**Acute treatment (%)**						
Reperfusion treatment	8.6	13.2	16.4	··	··	··
Iv thrombolysis	8.1	12.3	13.9	··	··	··
Thrombectomy	1.1	1.9	4.8	··	··	··
**Oral anticoagulants at admission (%)**						
Vitamin K antagonist (VKA)	6	6.9	5.3	12.4	15.1	11.7
Non-VKA (NOAC)	0.5	2.2	7.3	0.7	2.9	10.9

Abbreviations: AF = atrial fibrillation; ADL = activities of daily living; Iv = intravenous; NOAC = non-vitamin K antagonist oral anticoagulants.

Fewer patients were drowsy (RLS 2–3) or comatose (RLS 4–8) when admitted to hospital in 2017–2019 compared to 2010–2012 (11.3% vs 12.9% [*P* < .001] for IS and 36.8% vs 38.8% [*P* = .01] for ICH, respectively). The proportion of pre-stroke independent patients with IS increased from 76.6% in 2010–2012 to 77.6% in 2017–2019 (*P* < .001), but the proportion of pre-stroke independent patients with ICH decreased over the same time period (from 76.5% to 74.8%, *P* = .02). In patients with IS, acute reperfusion treatment (IVT and/or MT) increased from 8.6% in 2010–2012 to 16.4% in 2017–2019 (*P* < .001). Specifically, IVT increased from 8.1% to 13.9% (*P* < .001) and MT from 1.1% to 4.8% (*P* < .001), respectively. In patients with ICH, ongoing treatment with oral anticoagulants (OACs) at admission increased from 13.1% to 22.6% over the same time period (*P* < .001).

### Temporal changes in functional outcome

In 90-day stroke survivors (*n* = 148,721), data on functional outcome at 90 days were available for 82.0% of cases between 2010 and 2019. The proportion of cases lost to follow-up (missing or incomplete 90-day outcome data) was 14.7% for the 2010–2012 time period, 17.8% for the 2013–2016 time period, and 22.0% for the 2017–2019 time period (14.3%, 17.2% and 21.3% in patients with IS and 18.3%, 23.0% and 28.0% in patients with ICH, respectively). Patients lost to follow-up had baseline characteristics associated with less favourable outcomes, being more often dependent in ADL and more likely to present with reduced consciousness at admission (Table S3a). However, temporal changes in selected baseline characteristics among patients lost to follow-up were comparable to those observed in the overall study cohort (Table S3b and Table 1). As expected, multiple imputation to account for missing outcome data slightly increased the proportion of patients with poor functional outcome, although the overall distribution of mRS scores differed by only about 0.5%. Importantly, the observed changes between time periods remained largely consistent between analyses with and without imputed data (Table S4).

**Figure 1:**
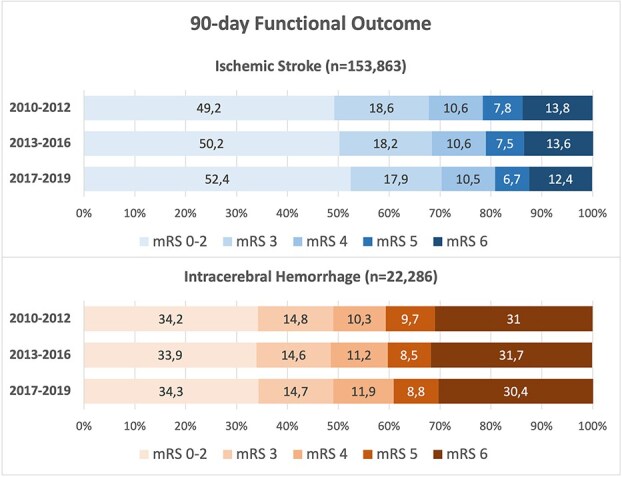
Crude data showing the distribution of functional outcome on the mRS after 90 days, for ischaemic stroke and ICH, including imputed data.

**Figure 2:**
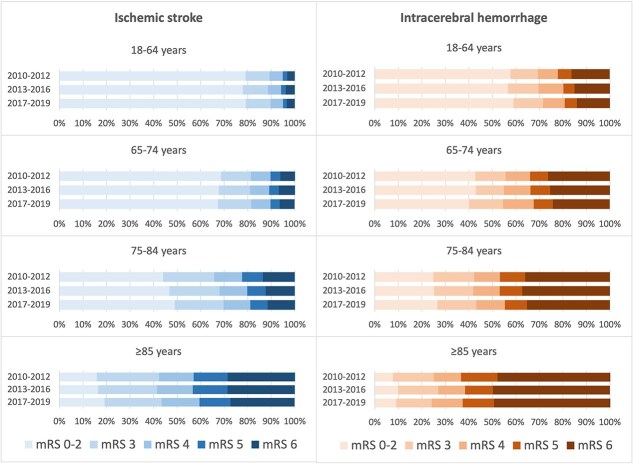
Unadjusted distribution of functional outcome on the mRS after 90 days, in patients with ischaemic stroke or ICH, for different age groups, including imputed data.

Figures 1 and 2 show the distribution of functional outcome on the mRS (0–2 to 6) after 90 days for patients with IS and ICH as *unadjusted Grotta bars for descriptive purpose*, including imputed data (the corresponding figure for mRS scores for IS and ICH combined are available as Figures S2 and S3). The results of the mRS at 90 days before multiple imputation (including proportions of missing mRS scores) are shown in Figure S4. *Adjusted associations are provided in the regression analyses.*

#### Ischaemic stroke

Good functional outcome (mRS 0–2) in patients with IS after 90 days increased significantly from 49.2% in 2010–2012 to 52.4% in 2017–2019. The corresponding adjusted OR (aOR) was 1.12 (95% CI, 1.09–1.16).

When analysing age-specific trends, an increase in good functional outcome was only observed in the older age groups of 75–84 years (from 50.9% in 2010–2012 to 55.5% in 2017–2019, aOR 1.19 [95% CI, 1.12–1.25]) and over 85 years (from 22.2% to 26.2%, aOR 1.25 [95% CI, 1.16–1.35]) (Figure 2, Table 2, Table S5 for IS and ICH combined).

**Table 2:** Crude and adjusted odds ratios for good functional outcome (mRS 0–2) after 90 days in survivors of ischaemic stroke and ICH for different age groups

	**Ischaemic stroke**			**Intracerebral haemorrhage**		
	**Year of stroke**			**Year of stroke**		
	**2010–2012**	**2013–2016**	**2017–2019**	**2010–2012**	**2013–2016**	**2017–2019**
**All ages**						
mRS 0–2, %	57.1	58.0	59.9	49.6	49.7	49.2
Crude OR (95% CI)	1 (reference)	1.04 (1.01–1.07)^a^	1.12 (1.09–1.15)^a^	1 (reference)	1.00 (0.93–1.08)	0.98 (0.91–1.07)
Adjusted OR (95% CI)	1 (reference)	1.04 (1.01–1.07)^a^	1.12 (1.09–1.16)^a^	1 (reference)	0.99 (0.91–1.08)	0.96 (0.88–1.06)
**18–64 years**						
mRS 0–2, %	81.8	81.1	82.0	69.0	66.6	68.7
Crude OR (95% CI)	1 (reference)	0.96 (0.89–1.03)	1.01 (0.94–1.10)	1 (reference)	0.90 (0.78–1.04)	0.99 (0.84–1.16)
Adjusted OR (95% CI)	1 (reference)	0.95 (0.88–1.02)	1.02 (0.94–1.11)	1 (reference)	0.88 (0.75–1.03)	0.98 (0.82–1.16)
**65–74 years**						
mRS 0–2, %	73.2	72.7	72.0	58.1	57.4	53.0
Crude OR (95% CI)	1 (reference)	0.98 (0.92–1.03)	0.94 (0.89–1.00)	1 (reference)	0.98 (0.84–1.14)	0.81 (0.69–0.96)^a^
Adjusted OR (95% CI)	1 (reference)	1.00 (0.94–1.06)	0.99 (0.93–1.06)	1 (reference)	0.95 (0.80–1.11)	0.81 (0.68–0.97)^a^
**75–84 years**						
mRS 0–2, %	50.9	53.3	55.5	38.9	40.3	41.3
Crude OR (95% CI)	1 (reference)	1.10 (1.05–1.15)^a^	1.20 (1.15–1.26)^a^	1 (reference)	1.06 (0.91–1.23)	1.11 (0.94–1.30)
Adjusted OR (95% CI)	1 (reference)	1.08 (1.02–1.13)^a^	1.19 (1.12–1.25)^a^	1 (reference)	1.01 (0.86–1.18)	1.03 (0.87–1.22)
**≥85 years**						
mRS 0–2, %	22.2	23.1	26.2	14.8	19.8	18.0
Crude OR (95% CI)	1 (reference)	1.05 (0.98–1.12)	1.25 (1.16–1.34)^a^	1 (reference)	1.42 (1.10–1.85)^a^	1.26 (0.95–1.68)
Adjusted OR (95% CI)	1 (reference)	1.08 (1.00–1.16)	1.25 (1.16–1.35)^a^	1 (reference)	1.40 (1.06–1.85)^a^	1.18 (0.87–1.60)

^a^Statistically significant (*P* < .05).

Adjusted for age, sex, stroke severity and pre-stroke independency.

Abbreviation: OR = odds ratio.

#### Intracerebral haemorrhage

The proportion of good functional outcome (mRS 0–2) in patients with ICH after 90 days did not change significantly from 34.2% in 2010–2012 to 34.3% in 2017–2019, corresponding to an aOR 0.96 (95% CI, 0.88–1.06).

We observed an increased proportion of good functional outcome in patients with ICH between 75 and 84 years (24.8% in 2010–2012 compared to 26.6% in 2017–2019 (aOR 1.03 [95% CI, 0.87–1.22]) and over 85 years from 7.7% to 9.1% (aOR 1.18 [95% CI, 0.87–1.60]). In contrast, there was a significantly decrease in good functional outcome in the 65–74 years age group from 42.8% in 2010–2012 to 40.1% in 2017–2019 (aOR 0.81 [95% CI, 0.68–0.97]) (Figure 2, Table 2, Table S5 for IS and ICH combined).

### Temporal changes in case-fatality at 90 days

Data on CF were available for all patients and are presented for IS and ICH separately. For the Cox regression analyses, the total follow-up corresponded to 12.48 million person-days for IS and 1.46 million person-days for ICH.

#### Ischaemic stroke

For patients with IS, 90-day CF decreased from 13.8% in 2010–2012 to 12.4% in 2017–2019 (*P* < .001) (as mRS 6 in Figure 1). The crude HR for death within 90 days was 0.89 (95% CI, 0.86–0.93). After adjustment for age, sex, stroke severity and pre-stroke independency, the adjusted HR for death was 0.99 (95% CI, 0.95–1.02) (Table 3).

**Table 3:** Crude and adjusted hazard ratios for 90-day case-fatality in patients with ischaemic stroke or ICH for different age groups

	**Ischaemic stroke**			**Intracerebral haemorrhage**		
	**Year of stroke**			**Year of stroke**		
	**2010–2012**	**2013–2016**	**2017–2019**	**2010–2012**	**2013–2016**	**2017–2019**
**All ages**						
90-day case fatality, %	13.8	13.6	12.4	31.0	31.7	30.4
Crude HR (95% CI)	1 (reference)	0.98 (0.95–1.01)	0.89 (0.86–0.93)^a^	1 (reference)	1.03 (0.97–1.08)	0.97 (0.91–1.03)
Adjusted HR (95% CI)	1 (reference)	1.03 (0.99–1.06)	0.99 (0.95–1.02)	1 (reference)	1.06 (1.00–1.12)^*^	1.00 (0.94–1.06)
**18–64 years**						
90-day case fatality, %	3.3	3.9	3.4	16.3	15.1	14.1
Crude HR (95% CI)	1 (reference)	1.18 (1.02–1.37)^a^	1.03 (0.88–1.21)	1 (reference)	0.92 (0.79–1.08)	0.86 (0.72–1.02)
Adjusted HR (95% CI)	1 (reference)	1.11 (0.96–1.28)	0.96 (0.82–1.14)	1 (reference)	1.00 (0.85–1.17)	0.91 (0.77–1.09)
**65–74 years**						
90-day case fatality, %	6.3	6.9	6.4	26.4	25.5	24.3
Crude HR (95% CI)	1 (reference)	1.10 (1.00–1.21)^a^	1.03 (0.93–1.14)	1 (reference)	0.95 (0.84–1.08)	0.90 (0.79–1.04)
Adjusted HR (95% CI)	1 (reference)	1.09 (0.99–1.20)	0.99 (0.89–1.10)	1 (reference)	1.01 (0.89–1.14)	0.86 (0.75–0.99)^a^
**75–84 years**						
90-day case fatality, %	13.6	12.4	11.7	36.1	37.4	35.4
Crude HR (95% CI)	1 (reference)	0.90 (0.85–0.96)^a^	0.85 (0.79–0.90)^a^	1 (reference)	1.05 (0.95–1.15)	0.97 (0.87–1.08)
Adjusted HR (95% CI)	1 (reference)	0.96 (0.90–1.02)	0.92 (0.86–0.98)^a^	1 (reference)	1.06 (0.96–1.17)	0.99 (0.89–1.10)
**≥85 years**						
90-day case fatality, %	28.7	28.7	27.3	48.0	49.8	49.5
Crude HR (95% CI)	1 (reference)	1.00 (0.96–1.05)	0.95 (0.91–1.00)	1 (reference)	1.06 (0.96–1.17)	1.05 (0.94–1.17)
Adjusted HR (95% CI)	1 (reference)	1.04 (0.99–1.08)	1.02 (0.97–1.07)	1 (reference)	1.14 (1.03–1.26)^a^	1.17 (1.05–1.31)^a^

^a^Statistically significant (*P* < .05).

Adjusted for age, sex, stroke severity and pre-stroke independency.

Abbreviation: HR, hazard ratio.

Case-fatality was lower in younger patients (<75 years CF 5.3%, ≥ 75 years CF 19.6%), but there was no improvement in CF from 2010–2012 to 2017–2019 in age-groups 18–64 years (CF from 3.3% to 3.4%, *P* = .64) and 65–74 years (6.3%–6.4%, *P* = .61), respectively. In the age-group 75–84 years, CF decreased from 13.6% in 2010–2012 to 11.7% in 2017–2019 (*P* < .01) and in patients over 85 years, CF decreased from 28.7% to 27.3% (*P* = .03), respectively. Adjusted HRs for death within 90 days were only significantly reduced for the age-group 75–84 years (adjusted hazard ratio [aHR] 0.92; 95% CI, 0.86–0.98) (as mRS 6 in Figure 2, Table 3, Table S6 for IS and ICH combined).

#### Intracerebral haemorrhage

In patients with ICH, 90-day CF remained largely unchanged, from 31.0% in 2010–2012 to 30.4% in 2017–2019 (*P* = .44) (as mRS 6 in Figure 1). The adjusted HR for death within 90 days was 1.00 (95% CI, 0.94–1.06) when comparing 2017–2019 to 2010–2012 (Table 3).

Case-fatality decreased in 18- to 64-year-olds (from 16.3% to 14.1%, *P* = .07) and in 65- to 74-year-olds (from 26.4% to 24.3%, *P* = .18) from 2010–2012 to 2017–2019. Adjusted HRs (aHR) indicated a significant decrease in CF over time in the age-group 65–74 years with aHR 0.86 (95% CI, 0.75–0.99). In contrast, CF increased from 48.0% in 2010–2012 to 49.5% in 2017–2019 in patients over 85 years (aHR 1.17; 95% CI, 1.05–1.31) (as mRS 6 in Figure 2, Table 3, Table S6 for IS and ICH combined).

## Discussion

Our study showed that the proportion of patients with first-time stroke having good functional outcome (mRS 0-2) at 90 days increased over the study period for IS (+3.2%), but not for ICH (+0.1%). For patients with IS, this improvement in functional outcome was seen in patients over 75 years. Case-fatality at 90 days decreased slightly in patients both with IS (−1.2%) and ICH (−0.6%) from 2010–2012 to 2017–2019, but this reduction was not significant after adjusting for relevant confounders.

### Temporal changes in functional outcome

Improvements in functional outcome over time following IS have previously been reported in studies from England, Canada and Japan.^7–9^ However, comparing our results with these studies in terms of magnitude of improvement is difficult because of different outcome scales/parameters and time points for the estimation of functional outcome. The Japanese study used the mRS score to measure functional outcome, but at discharge from hospital. For the time periods 2011–2015 and 2016–2019, they reported proportions of patients with mRS 0–2 in 48% of women and 62% of men, which is slightly higher than in our cohort (about 41% in women and 59% in men).^8^ The increase in good functional outcome over time in the Japanese cohort remained significant after adjusting for age, but not after multivariate adjustments for stroke severity and reperfusion treatment. In our cohort, there was a significant improvement in good functional outcome after adjusting for age, sex, stroke severity and pre-stroke functional status. The study from England examined trends in functional dependence 3 months after IS using the Barthel Index as a measure of functional outcome. They also reported the proportion of patients with mRS ≥ 3 derived from Barthel Index scores. Their results showed a decline in functionally dependent patients (mRS ≥ 3) among IS survivors from 43.6% in 2000–2003 to 35.1% in 2012–2015.^7^ In our study, the proportion of IS survivors with poor functional outcome (mRS 3–5) decreased from 42.9% in 2010–2012 to 40.1% in 2017–2019. Although the time periods differ, the proportions of poor functional outcome in our study are slightly higher than those reported in the English cohort. This difference is likely attributable to the higher mean age in our cohort (76–77 years vs 69–72 years in the English cohort). A recent study from Auckland, New Zealand, reported trends in stroke outcome over 4 decades using the mRS score. However, a trend analysis was not performed for IS and ICH separately. They showed an increase in the proportion of good functional outcome (mRS 0–2) over time, but also an increase in the proportion of poor functional outcome (mRS 3–5), which might be linked to decreased CF over the same time period.^10^

One explanation for the improvement in functional outcome after IS seen in our study could be a trend towards less severe strokes which has previously been shown in registries from Austria, Israel and Japan.^8^^,^^21^^,^^22^ In our study, we used level of consciousness at admission as a proxy for stroke severity. We observed a slight but significant decreased proportion of severe stroke (drowsy or comatose at hospital admission) from 2010–2012 to 2017–2019, which reasonably might influence changes over time in functional outcome. When adjusting our results for stroke severity, the OR for good functional outcome was still significant when comparing 2017–2019 with 2010–2012. Reperfusion treatments improve functional outcome after IS.^23^ In our study, the proportion of patients receiving reperfusion treatments nearly doubled from 8.6% in 2010–2012 to 16.4% in 2017–2019, which could be an additional explanation for the observed improvement in functional outcome. However, the trend for improvement of functional outcome in IS remained significant after adjusting for reperfusion treatment in the logistic regression analysis. Early stroke recurrence within 90 days may negatively impact functional outcomes. As the risk of recurrent stroke declined in Sweden between 2012 and 2019–2020,^24^ the improvement in functional outcomes observed in our study may be partly attributable to this trend.

An increased proportion of patients with good functional outcome after IS was mainly observed in patients over 75 years. In contrast to our results, a study from New Zealand showed an improvement in good functional outcome for patients under 75 years from 1981–1982 to 2021–2022.^10^ However, comparing these data to our results is limited due to the vastly different time span. One possible explanation for the positive changes in functional outcome seen in older patients in Sweden could be a general improvement in the overall health status of the elderly population.^25^^,^^26^ Another explanation may be the increased use of OAC in patients with AF, which could have prevented more severe cardioembolic IS caused by large cardiac thrombi.^21^ Since the prevalence of AF increases with age, this preventive effect would be particularly relevant in older patients. Supporting this, a Japanese observational study recently demonstrated a beneficial effect of OAC therapy on stroke severity in patients over 75 years.^27^ In addition, our own data show that the proportions of reperfusion treatment increased more markedly in patients over 75 years (from 5.9% in 2010–2012 to 15.0% in 2017–2019), compared to a smaller increase in patients under 75 years (from 12.4% to 18.1%). This greater improvement in access to reperfusion therapies among older patients could also have contributed to the more favourable changes in functional outcomes over time observed in this group.

Beyond advances in acute treatment and secondary prevention, broader contextual factors may also have contributed to the observed trends. Increased public awareness and improved stroke recognition may have led to earlier hospital presentation and treatment initiation.^28^ In addition, national quality improvement initiatives and policy-driven expansion of stroke unit care could have strengthened overall management and outcomes. When comparing our results with other high-income countries, differences in healthcare organisation, timing of policy implementation, and access to specialised stroke care should be considered. However, our study was not designed to assess the relative impact of these factors on the observed trends, which should be addressed in future studies.

Functional outcome after ICH did not improve over time in our study. This is in line with a recent review and meta-analysis which reported no trends in functional outcome over time after ICH.^4^ In this meta-analysis, the percentage of patients with good functional outcome after 3–12 months was 31.2%, which is slightly lower than our results (34% with mRS 0–2). Between 2010–2012 and 2017–2019, we observed an increased proportion of patients with OAC-associated ICH (from 13.1% to 22.5%). This could be a possible explanation for the neutral trend in functional outcome after ICH observed in our study, since OAC-associated ICH have worse functional outcome.^29^

### Trends in case-fatality

In our study, overall 90-day CF in patients with IS between 2010 and 2019 was around 13%, which is comparable with results from a population-based study from Germany (3-month CF 13% in 1996–2015)^30^ and a regional study from Canada (30-day CF around 12% in 2010–2017).^9^ The slight decrease in CF after IS from 2010–2012 to 2017-2019 observed in our study was no longer significant after adjusting for changes over time in age, sex, stroke severity and pre-stroke independency. In 2017–2019 compared to 2010–2012, patients in the IS cohort had a lower median age, a higher proportion of males, less severe strokes, and a lower prevalence of pre-stroke dependency. These factors likely contributed to a reduced risk of fatal IS and may explain the observed decrease in crude CF after IS. However, in patients 75–84 years of age, a lower risk of death within 90 days after IS remained significant after adjusting for age, sex, stroke severity and pre-stroke independency, likely implicating other factors contributing to a decrease in CF in these older patients. In contrast to our results, studies from Canada and England did show a significant decrease in CF after IS.^7^^,^^9^ One possible explanation could be that these studies analysed longer time periods and included earlier years (Canada: 2003–2017; England: 2000–2015) than our study.

Overall 90-day CF after first-ever ICH was 31% in our study, which is lower compared to recently published results from a meta-analysis reporting a 1-month CF after ICH of 34.8% in high-income countries.^4^ The difference might, in part, be explained by a higher CF in recurrent ICH which were not excluded in the meta-analysis. Additionally, CF after ICH showed large variations between different countries and the meta-analysis included older studies ranging from 1982 to 2019.

Even if there was a minimal decrease in crude CF after ICH in our study, changes over time were not significant in either the unadjusted or adjusted analyses, which is in line with results from the meta-analysis.^4^ Possible explanations are assumably with the same as the reasons for stable patterns over time in functional outcome after ICH as discussed above. In patients over 85 years, we observed a significantly higher risk of death within 90 days in 2017–2019 compared to 2010–2012. In this age group, the proportion of OAC-associated ICH increased from 14.3% to 38.2% over the study period, which could explain this result.

### Strengths and limitations

The strength of our study is the nationwide design and the large number of patients with first-ever IS (about 154,000) and first-ever ICH (about 22,000). Another strength is the consistently high coverage of Riksstroke, capturing approximately 90% of all hospitalised stroke cases in Sweden when compared with the Swedish National Patient Register, and about 82% of all stroke cases when compared with a population-based cohort.^31^ The remaining cases are primarily patients with high early case-fatality rates, mild strokes or those residing in nursing care facilities. However, the coverage rate and potential selection biases were likely stable throughout the study period, supporting the validity of conclusions regarding temporal changes.

Our study also has several limitations. The proportion of patients lost to follow-up (15.2%) due to missing or incomplete data required for estimating mRS scores is an important limitation. The proportion of patients lost to follow-up increased during the study period from 12.4% in 2010–2012 to 18.2% in 2017–2019. This trend likely reflects a general decline in survey response rates in Sweden.^32^ However, changes over time in baseline characteristics among patients lost to follow-up were comparable to those observed in the overall study cohort. We used multiple imputation to estimate mRS scores in those lost to follow-up based on their baseline characteristics and used outcome data from comparable patients with available information. Excluding patients with missing outcome data from our analysis could have led to an underestimation of the proportion of patients with poor functional outcome. Patients with missing outcome data were more likely to be pre-stroke dependent and presented with more severe strokes (Table S3a). To assess the impact of imputation, a complete-case sensitivity analysis was conducted, which produced results largely consistent with the imputed data, thereby reinforcing the robustness of our findings. Nonetheless, incomplete longitudinal data on functional outcome are a common challenge in large cohort studies, and optimal approaches for handling such data remain an area of ongoing methodological research. The NIHSS would have provided more detailed information on stroke severity than level of consciousness, especially in less severe stroke, but values for NIHSS scores were missing in about 50% of patients. However, level of consciousness has been previously demonstrated as a good proxy for the full NIHSS in predicting mortality.^33^ While information on smoking was available, data on other lifestyle factors such as alcohol consumption, diet, and physical activity were not collected and could therefore not be included in the analyses. Due to the observational study design, residual confounding may remain despite adjustment for key covariates. Finally, although functional outcome was assessed using a validated algorithm translating indicators such as dressing, toileting, and mobility into mRS scores, potential limitations related to this transformation should be considered.

## Conclusion

We observed improvement in functional outcome after first-ever IS, but not after first-ever ICH, in Sweden between 2010 and 2019. Possible explanations for the positive trend in IS are a decline in stroke severity, improvement in acute stroke care, or a combination of these. Remarkably, changes over time in functional outcome were more favourable in older patients and further analysis is needed to identify the underlying causes for this observation. Case-fatality decreased in both patients with IS and ICH during the study period, but the reduction was not significant after adjustment for confounding.

Our results highlight that despite the observed improvements in functional outcome after IS during our study period, the overall prognosis after stroke remains serious and further efforts are needed to reduce the burden of acute stroke.

## Supplementary Material

aakaf021_RECORD_Checklist_Conrad_Drescher_ESJ_resubmission

aakaf021_Supplementary_material_ESJ_resubmission

aakaf021_Drescher_25-0614_VA

## Data Availability

An aggregated dataset may be shared upon reasonable request, including necessary approvals.
