# Supplementary material for: Temporal changes in functional outcome and case-fatality after ischaemic stroke and intracerebral haemorrhage in Sweden 2010–2019: an observational study from the Swedish Stroke Register (Riksstroke)
Source: Eur Stroke J. 2026 Jan 1;11(1):aakaf021. doi: 10.1093/esj/aakaf021 (PMC12866662; doi:10.1093/esj/aakaf021)
Supplement: aakaf021_Supplementary_material_ESJ_resubmission [file aakaf021_supplementary_material_esj_resubmission.docx]

**Supplementary Material**

**Appendix**

Supplementary Figure S1………………................................……………………………...2

- Study flow chart

Supplementary Table S1…………………………………………………………………….3

- Baseline Characteristics for ischemic stroke and intracerebral hemorrhage combined

Supplementary Table S2…………………………………………………………………….4

- Baseline Characteristics for unspecified stroke (I64)

Supplementary Table S3a..………………………………………………………………….5

- Selected baseline variables for patients followed up and lost to follow-up

Supplementary Table S3b..………………………………………………………………….5

- Selected baseline variables for patients lost to follow-up for different time periods

Supplementary Table S4....………………………………………………………………….6

- comparison between original and imputed data sets for mRS scores in survivors in ischemic stroke and intracerebral hemorrhage for 3 time periods

Supplementary Figure S2…………………………………………………………………...7

- mRS distribution in ischemic stroke and intracerebral hemorrhage combined for all ages (with imputated data)

Supplementary Figure S3…………………………………………………………………...8

- mRS distribution in ischemic stroke and intracerebral hemorrhage combined for different age groups (with imputated data)

Supplementary Figure S4…………………………………………………………………...9

- mRS distribution in ischemic stroke and intracerebral hemorrhage combined including proportions of missing data

Supplementary Table S5……………………………………………………………………10

- odds ratios for good functional outcome in ischemic stroke and intracerebral hemorrhage combined

Supplementary Table S6……………………………………………………………………11

- hazarad ratios for 90-day case-fatality in ischemic stroke and intracerebral hemorrhage combined

Supplementary Table S7………………………………………………………………..12–13

- odds ratios for good functional outcome in ischemic stroke and intracerebral hemorrhage in patient follow-up (without imputed data)

**Supplementary Figure S1:** Study flow chart

**Riksstroke events**

**2010–2019**

IS+ICH: n=230,577

IS: n=201,315 (87.3%)

ICH: n=29,262 (12.7%)

**Cases with previous stroke**

IS+ICH: n=54,428

IS: n=47,452

ICH: n=6,976

excluded

**First-ever events**

IS+ICH: n=176,149

IS: n=153,863 (87.3%)

ICH: n=22,286 (12.7%)

**Alive at 90 days and followed up**

IS+ICH n=121,919 (69.2%)

IS n=110,107 (71.6%)

ICH n=11,812 (53.0%)

**Dead at 90 days**

IS+ICH n=27,428 (15.6%)

IS n=20,494 (13.3%)

ICH n=6,934 (31.1%)

**Alive at 90 days and lost to follow up**

IS+ICH n=26,802 (15.2%)

IS n=23,262 (15.1%)

ICH n=3,540 (15.9%)

**Supplementary Table S1:** Patient characteristics for first-ever ischemic stroke and first-ever intracerebral hemorrhage combined in 3 time periods.

|  | **Ischemic Stoke + Intracerebral Hemorrhage** | | |
| --- | --- | --- | --- |
|  | **2010–2012** | **2013–2016** | **2017–2019** |
|  | N=55,673 | N=70,635 | N=49,846 |
|  |  |  |  |
| Cases per year n | 18,558 | 17,659 | 16,615 |
| Male sex (%) | 51.0 | 51.8 | 53.2 |
| Age as median (IQR) | 77 (67–85) | 76 (67–85) | 76 (67–84) |
| **Risk factors (%)** | | | |
| Hypertension | 56.7 | 58.7 | 60.0 |
| Diabetes | 18.8 | 19.8 | 21.5 |
| Smoking | 13.7 | 13.5 | 12.7 |
| AF total | 26.4 | 27.2 | 27.1 |
| Previous TIA | 7.0 | 7.1 | 7.4 |
| **Stroke severity (%)** | | | |
| Alert | 82.8 | 83.5 | 83.9 |
| Drowsy | 11.5 | 10.7 | 10.5 |
| Unconscious | 4.5 | 4.5 | 4.2 |
| **Premorbid status (%)** | | | |
| Institutional Living | 7.0 | 7.3 | 6.7 |
| ADL-independency | 76.6 | 76.5 | 77.2 |
| **Medication at admission (%)** | | | |
| Vitamin K antagonist (VKA) | 6.7 | 8.0 | 6.1 |
| Non-VKA (NOAC) | 0.5 | 2.3 | 7.7 |

IQR, interquartile range; AF, atrial fibrillation; TIA, transient ischemic attack;

ADL, activities of daily living; NOAC, non-vitamin K antagonist oral anticoagulants

|  | **First-ever unspecified stroke (I64)** | | |
| --- | --- | --- | --- |
|  | **2010–2012** | **2013–2016** | **2017–2019** |
|  | N=814 | N=769 | N=475 |
|  |  |  |  |
| Cases per year n | 271 | 192 | 158 |
| % of all stroke cases | 1.4 | 1.1 | 0.9 |
| Male sex (%) | 43.2 | 46.2 | 48.2 |
| Age as median (IQR) | 82 (73–88) | 81 (72–88) | 80 (72–87) |
| **Risk factors (%)** | | | |
| Hypertension | 54.8 | 62.0 | 62.1 |
| Diabetes | 19.9 | 17.8 | 23.8 |
| Smoking | 9.1 | 9.8 | 8.8 |
| AF total | 30.8 | 27.8 | 30.1 |
| Previous TIA | 7.1 | 7.3 | 9.9 |
| **Stroke severity (%)** | | | |
| Alert | 73.5 | 77.0 | 76.0 |
| Drowsy | 14.7 | 11.2 | 12.2 |
| Unconscious | 10.8 | 9.0 | 8.4 |
| **Premorbid status (%)** | | | |
| Institutional Living | 18.9 | 13.9 | 14.5 |
| ADL-independency | 57.1 | 62.2 | 61.1 |
| **Acute treatment (%)** | | | |
| Reperfusion treatment | 1.7 | 2.0 | 0.2 |
| Iv thrombolysis | 1.5 | 1.8 | 0.0 |
| Thrombectomy | 0.2 | 0.1 | 0.2 |
| **Oral anticoagulants at admission (%)** | | | |
| Vitamin K antagonist (VKA) | 6.3 | 7.5 | 5.3 |
| Non-VKA (NOAC) | 0.4 | 2.7 | 8.6 |

**Supplementary Table S2:** Patient characteristics for first-ever unspecified stroke (I64) for 3 different time periods

IQR, interquartile range; AF, atrial fibrillation; TIA, transient ischemic attack;

ADL, activities of daily living; Iv, intravenous; NOAC, non-vitamin K antagonist oral anticoagulants

**Supplementary Table S3a**: Selected baseline characteristics in patients alive and followed up, alive and lost to follow up and dead at 90 days after ischemic stroke and intracerebral hemorrhage combined.

|  | **Alive at 90 days and followed up**  N=121,919 (69.2%) | **Alive at 90 days and lost to follow-up**  N=26,802 (15.2%) | **Dead at 90 days**  N=27,428 (15.6) |
| --- | --- | --- | --- |
| Male Sex (%) | 53.6 | 52.5 | 43.8 |
| Age as median (IQR) | 75 (66-83) | 75 (63-84) | 84 (76-89) |
| Diagnosis IS (%) | 90.3 | 86.8 | 74.7 |
| ADL independency (%) | 83.9 | 73.2 | 48.3 |
| Level of consciousness (%) |  |  |  |
| Alert | 91.8 | 86.1 | 48.1 |
| Drowsy | 6.9 | 10.9 | 30.9 |
| Comatose | 1.3 | 3.0 | 21.0 |

IQR, interquartile range; IS, ischemic stroke; ADL, activities of daily living

**Supplementary Table S3b**: Selected baseline characteristics in patients alive and lost to follow up after ischemic stroke and intracerebral hemorrhage combined for the 3 different time periods.

|  | **Alive at 90 days and lost to follow-up** | | |  |
| --- | --- | --- | --- | --- |
|  | **2010–2012**  N=6,885 (14.7%) | **2013–2016**  N=10,558 (17.8%) | **2017–2019**  N=9,359 (22.0%) | **P-value** |
| Male Sex (%) | 51.1 | 51.8 | 54.4 | **<0.001** |
| Age as median (IQR) | 75 (63-84) | 74 (64-84) | 74 (64-83) | **<0.001** |
| Diagnosis IS (%) | 87.7 | 86.4 | 86.5 | **0.04** |
| ADL independency (%) | 73.6 | 73.1 | 73.1 | 0.53 |
| Level of consciousness (%) |  |  |  | 0.64 |
| Alert | 85.6 | 86.3 | 86.2 |  |
| Drowsy | 11.6 | 10.7 | 10.7 |  |
| Comatose | 2.8 | 3.0 | 3.0 |  |

IQR, interquartile range; IS, ischemic stroke; ADL, activities of daily living

**Supplementary Table S4.** Proportion of mRS scores in survivors in ischemic stroke and intracerebral hemorrhage for 3 time periods, comparison between original and imputed data sets. In the original data set, cases lost to follow-up were omitted. In the imputed data sets, missing data were replaced with imputed values. Five imputations were performed, and a mean was calculated. The mean as well as lowest and highest proportion in the five individual imputations are presented.

|  | **Ischemic Stroke** | | | | | | | | | | | |
| --- | --- | --- | --- | --- | --- | --- | --- | --- | --- | --- | --- | --- |
|  | **Original data (n=110,110)** | | | **Imputed data (n=133,371)** | | | | | | | | |
|  | 2010–12 | 2013–16 | 2017–19 | 2010–12 | | | 2013–16 | | | 2017–19 | | |
|  |  |  |  | Mean | Lowest | Highest | Mean | Lowest | Highest | Mean | Lowest | Highest |
| **mRS 0–2** | 57.3 | 58.2 | 60.2 | 57.1 | 57.0 | 57.1 | 58.0 | 58.0 | 58.1 | 59.9 | 59.8 | 60.0 |
| **mRS 3** | 21.2 | 20.8 | 20.2 | 21.6 | 21.5 | 21.8 | 21.0 | 21.0 | 21.1 | 20.5 | 20.4 | 20.6 |
| **mRS 4** | 13.0 | 13.0 | 12.8 | 12.3 | 12.2 | 12.4 | 12.3 | 12.3 | 12.4 | 12.0 | 11.9 | 12.1 |
| **mRS 5** | 8.5 | 8.0 | 6.9 | 9.0 | 9.0 | 9.1 | 8.6 | 8.5 | 8.7 | 7.7 | 7.6 | 7.8 |
|  | **Intracerebral Hemorrhage** | | | | | | | | | | | |
|  |  |  |  |  |  |  |  |  |  |  |  |  |
|  | **Original data (n=11,814)** | | | **Imputed data (n=15,355)** | | | | | | | | |
|  | 2010–12 | 2013–16 | 2017–19 | 2010–12 | | | 2013–16 | | | 2017–19 | | |
|  |  |  |  | Mean | Lowest | Highest | Mean | Lowest | Highest | Mean | Lowest | Highest |
| **mRS 0–2** | 47.3 | 46.9 | 45.5 | 49.6 | 49.4 | 49.9 | 49.7 | 49.3 | 50.0 | 49.2 | 48.9 | 49.6 |
| **mRS 3** | 21.7 | 21.5 | 21.5 | 21.4 | 21.2 | 21.6 | 21.4 | 20.9 | 21.8 | 21.1 | 20.5 | 21.7 |
| **mRS 4** | 17.9 | 19.8 | 20.7 | 15.0 | 14.8 | 15.3 | 16.4 | 16.0 | 16.9 | 17.1 | 16.9 | 17.4 |
| **mRS 5** | 13.1 | 11.7 | 12.3 | 14.0 | 14.0 | 14.1 | 12.5 | 12.3 | 12.6 | 12.6 | 12.4 | 12.8 |

**Supplementary Figure S2**: Crude data showing the distribution of functional outcome on the modified Rankin Scale (mRS) after 90 days, for ischemic stroke and intracerebral hemorrhage combined, including imputed data.

**Supplementary Figure S3**: Unadjusted distribution of functional outcome on the modified Rankin Scale (mRS) after 90 days, for ischemic stroke and intracerebral hemorrhage combined and for different age groups (including imputed data).


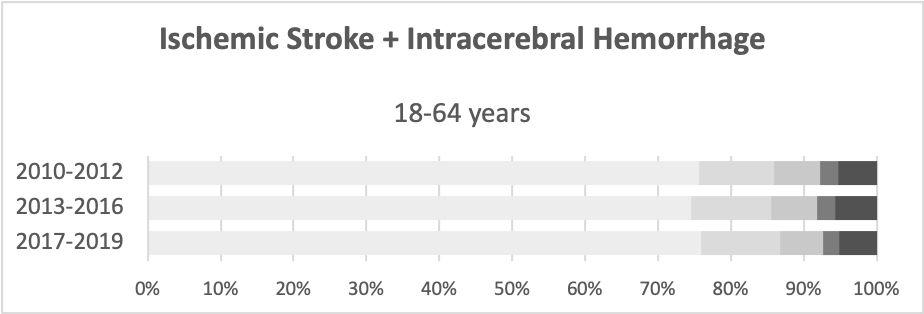

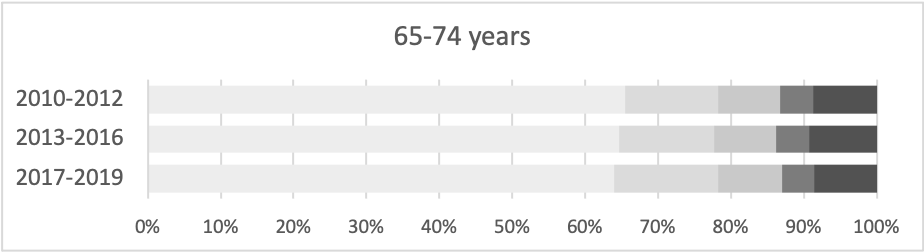

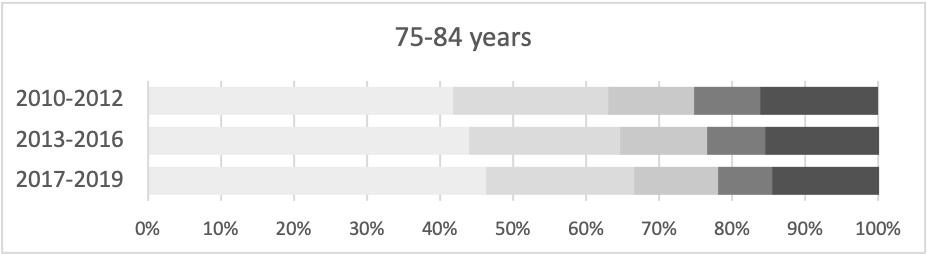

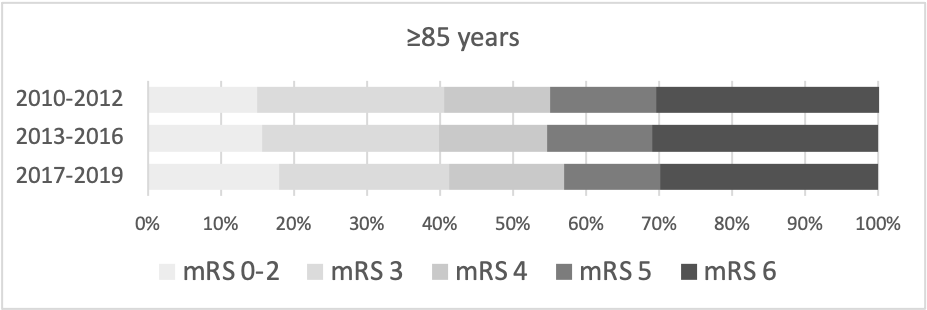


**Supplementary Figure S4**: Crude data showing the distribution of functional outcome on the modified Rankin Scale (mRS) after 90 days, for ischemic stroke and intracerebral hemorrhage combined without imputed data (showing proportions of missing data).


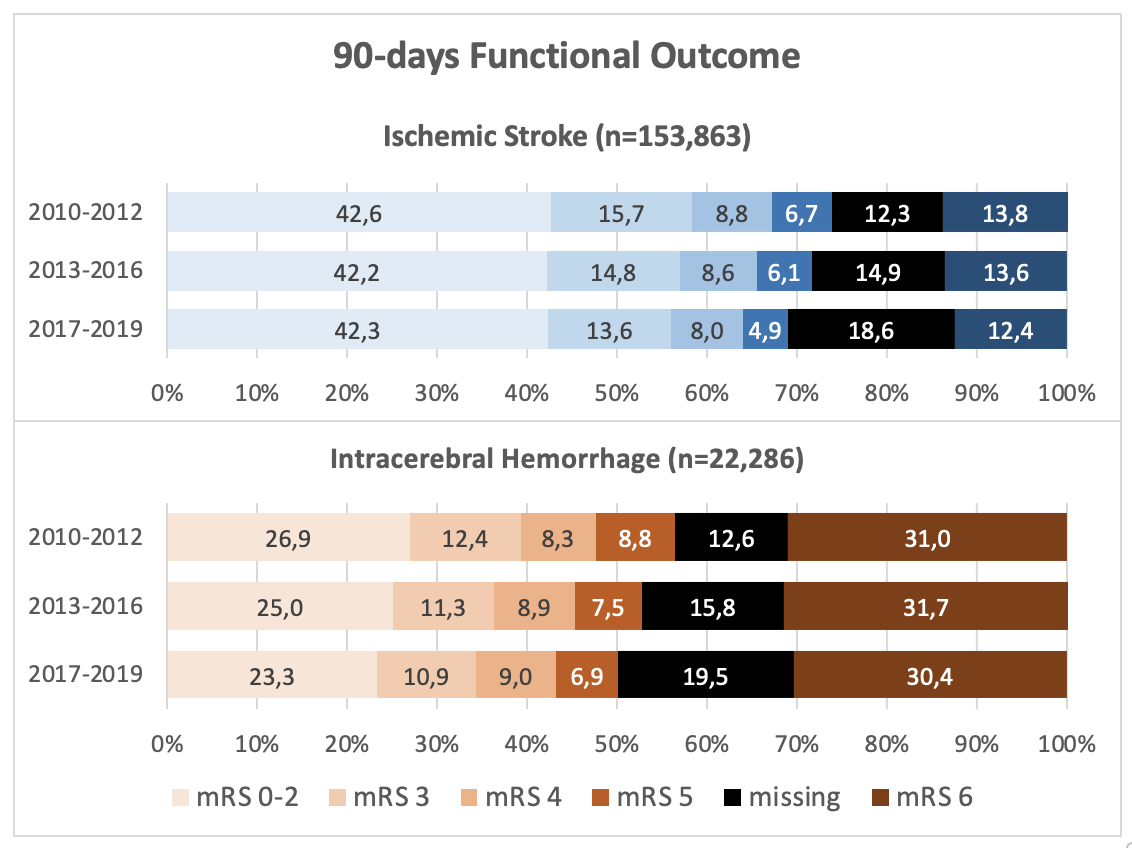


**Supplementary Table S5:** Crude and adjusted odds ratios for good functional outcome

(mRS 0–2) in stroke survivors for ischemic stroke and intracerebral hemorrhage combined and for different age groups.

|  | Year of stroke | | |
| --- | --- | --- | --- |
|  | **2010–2012** | **2013–2016** | **2017–2019** |
|  | **Ischemic Stroke + Intracerebral hemorrhage** | | |
| All ages | | | |
| mRS 0-2, % | 56.3 | 57.2 | 58.7 |
| Crude OR (95% CI) | 1 (reference) | 1.03 (1.01–1.06)* | 1.10 (1.07–1.13)* |
| Adjusted OR (95% CI) | 1 (reference) | 1.03 (1.00–1.06) | 1.10 (1.07–1.13)* |
|  | | | |
| 18–64 years | | | |
| mRS 0-2, % | 80.0 | 79.0 | 80.0 |
| Crude OR (95% CI) | 1 (reference) | 0.94 (0.89–1.01) | 1.00 (0.94–1.07) |
| Adjusted OR (95% CI) | 1 (reference) | 0.93 (0.87–1.00) | 1.01 (0.93–1.09) |
| 65–74 years | | | |
| mRS 0-2, % | 71.7 | 71.1 | 70.0 |
| Crude OR (95% CI) | 1 (reference) | 0.97 (0.92–1.03) | 0.92 (0.87–0.98)* |
| Adjusted OR (95% CI) | 1 (reference) | 0.99 (0.94–1.05) | 0.96 (0.91–0.02) |
| 75–84 years | | | |
| mRS 0-2, % | 49.9 | 52.1 | 54.2 |
| Crude OR (95% CI) | 1 (reference) | 1.09 (1.05–1.14)* | 1.19 (1.14–1.25)* |
| Adjusted OR (95% CI) | 1 (reference) | 1.07 (1.02–1.12)* | 1.17 (1.11–1.23)* |
| ≥85 years | | | |
| mRS 0-2, % | 21.6 | 22.8 | 25.5 |
| Crude OR (95% CI) | 1 (reference) | 1.07 (1.00–1.14)* | 1.24 (1.16–1.33)* |
| Adjusted OR (95% CI) | 1 (reference) | 1.09 (1.02–1.17)* | 1.24 (1.15–1.34)* |

Adjusted for age, sex, stroke severity and pre-stroke independency

* statistically significant (p<0.05)

OR, odds ratio; mRS, modified Rankin Scale; CI, confidence interval

**Supplementary Table S6:** Crude and adjusted hazard ratios for 90-day case-fatality for

ischemic stroke and intracerebral hemorrhage combined and for different age groups.

|  | Year of stroke | | |
| --- | --- | --- | --- |
|  | **2010–2012** | **2013–2016** | **2017–2019** |
|  | **Ischemic Stroke + Intracerebral hemorrhage** | | |
| All ages | | | |
| 90-day case fatality, % | 15.9 | 15.9 | 14.7 |
| Crude HR (95% CI) | 1 (reference) | 1.00 (0.98–1.03) | 0.93 (0.90–0.95)* |
| Adjusted HR (95% CI) | 1 (reference) | 1.05 (1.02–1.08)* | 1.01 (0.98–1.04) |
|  | | | |
| 18–64 years | | | |
| 90-day case fatality, % | 5.3 | 5.7 | 5.1 |
| Crude HR (95% CI) | 1 (reference) | 1.07 (0.96–1.19) | 0.96 (0.85–1.08) |
| Adjusted HR (95% CI) | 1 (reference) | 1.03 (0.93–1.15) | 0.93 (0.82–1.05) |
| 65–74 years | | | |
| 90-day case fatality, % | 8.7 | 9.2 | 8.7 |
| Crude HR (95% CI) | 1 (reference) | 1.06 (0.98–1.14) | 1.00 (0.92–1.08) |
| Adjusted HR (95% CI) | 1 (reference) | 1.07 (0.99–1.16) | 0.95 (0.87–1.03) |
| 75–84 years | | | |
| 90-day case fatality, % | 16.2 | 15.6 | 14.6 |
| Crude HR (95% CI) | 1 (reference) | 0.96 (0.91–1.01) | 0.89 (0.85–0.94)* |
| Adjusted HR (95% CI) | 1 (reference) | 1.01 (0.96–1.06) | 0.95 (0.90–1.01) |
| ≥85 years | | | |
| 90-day case fatality, % | 30.6 | 31.0 | 29.9 |
| Crude HR (95% CI) | 1 (reference) | 1.02 (0.98–1.07) | 0.99 (0.94–1.03) |
| Adjusted HR (95% CI) | 1 (reference) | 1.06 (1.02–1.11)* | 1.06 (1.01–1.11)* |

Adjusted for age, sex, stroke severity and pre-stroke independency

* statistically significant (p<0.05)

HR, hazard ratio; CI, confidence interval

**Supplementary Table S7:** Crude and adjusted odds ratios for good functional outcome (mRS 0–2) after 90 days in **survivors with complete follow up (patients lost to follow-up excluded)** of ischemic stroke and intracerebral hemorrhage for different age groups.

|  | Ischemic Stroke | | | Intracerebral Hemorrhage | | |
| --- | --- | --- | --- | --- | --- | --- |
|  | **Year of stroke** | | | **Year of stroke** | | |
|  | **2010–2012** | **2013–2016** | **2017–2019** | **2010–2012** | **2013–2016** | **2017–2019** |
| All ages | | | | | | |
| mRS 0–2, % | 57.7 | 58.9 | 61.4 | 47.7 | 47.7 | 46.5 |
| Crude OR (95% CI) | 1 (reference) | 1.05 (1.02–1.08)* | 1.17 (1.13–1.20)* | 1 (reference) | 1.00 (0.91–1.08) | 0.97 (0.88–1.06) |
| Adjusted OR (95% CI) | 1 (reference) | 1.05 (1.01–1.08)* | 1.15 (1.10–1.19)* | 1 (reference) | 0.94 (0.85–1.03) | 0.89 (0.80–0.99)* |
|  | | | | | | |
| 18–64 years | | | | | | |
| mRS 0–2, % | 81.9 | 80.8 | 82.1 | 65.5 | 62.2 | 61.8 |
| Crude OR (95% CI) | 1 (reference) | 0.94 (0.87–1.02) | 1.01 (0.92–1.11) | 1 (reference) | 0.84 (0.71–0.99)* | 0.89 (0.74–1.07) |
| Adjusted OR (95% CI) | 1 (reference) | 0.92 (0.84–1.00) | 0.99 (0.89–1.09) | 1 (reference) | 0.79 (0.66–0.95)* | 0.83 (0.68–1.01) |
| 65–74 years | | | | | | |
| mRS 0–2, % | 74.2 | 74.1 | 74.4 | 57.4 | 56.9 | 51.7 |
| Crude OR (95% CI) | 1 (reference) | 0.99 (0.93–1.05) | 1.01 (0.94–1.08) | 1 (reference) | 0.99 (0.84–1.17) | 0.80 (0.67–0.96)* |
| Adjusted OR (95% CI) | 1 (reference) | 1.01 (0.95–1.08) | 1.04 (0.97–1.12) | 1 (reference) | 0.92 (0.76–1.10) | 0.76 (0.62–0.93)* |
| 75–84 years | | | | | | |
| mRS 0–2, % | 51.9 | 54.9 | 57.6 | 38.7 | 40.1 | 40.9 |
| Crude OR (95% CI) | 1 (reference) | 1.13 (1.08–1.19)* | 1.26 (1.19–1.33)* | 1 (reference) | 1.06 (0.90–1.25) | 1.10 (0.92–1.32) |
| Adjusted OR (95% CI) | 1 (reference) | 1.10 (1.04–1.16)* | 1.20 (1.13–1.28)* | 1 (reference) | 0.99 (0.82–1.18) | 0.98 (0.81–1.19) |
| ≥85 years | | | | | | |
| mRS 0–2, % | 21.7 | 22.9 | 26.9 | 12.3 | 16.5 | 16.7 |
| Crude OR (95% CI) | 1 (reference) | 1.07 (1.00–1.15) | 1.32 (1.22–1.43)* | 1 (reference) | 1.42 (1.03–1.95)* | 1.44 (1.01–2.04)* |
| Adjusted OR (95% CI) | 1 (reference) | 1.08 (1.00–1.18) | 1.27 (1.16–1.39)* | 1 (reference) | 1.29 (0.92–1.82) | 1.21 (0.83–1.76) |

Adjusted for age, sex, stroke severity and pre-stroke independency

* statistically significant (p<0.05)

OR, odds ratio; mRS, modified Rankin Scale; CI, confidence interval
