# Supplementary material for: Temporal changes in functional outcome and case-fatality after ischaemic stroke and intracerebral haemorrhage in Sweden 2010–2019: an observational study from the Swedish Stroke Register (Riksstroke)
Source: Eur Stroke J. 2026 Jan 1;11(1):aakaf021. doi: 10.1093/esj/aakaf021 (PMC12866662; doi:10.1093/esj/aakaf021)
Supplement: aakaf021_Drescher_25-0614_VA [file aakaf021_drescher_25-0614_va.pdf]

EUROPEAN STROKE JOURNAL

Temporal changes in functional outcome and case-fatality after ischemic stroke and intracerebral hemorrhage in Sweden 2010–2019

To determine temporal changes in functional outcome and case-fatality 90 days after ischemic stroke (IS) and intracerebral hemorrhage (ICH) in Sweden 2010–2019

Methods

Nationwide, register-based observational study (Riksstroke)
First-ever IS or ICH ≥ 18 years

Functional outcome (mRS) and case-fatality (all-cause) at 90 days
Multiple imputation in lost to follow-up cases

Logistic regression (mRS 0–2) and Cox regression (death ≤ 90 days)
Reference period 2010–2012 (adjusted for confounding)

Results

Cohort

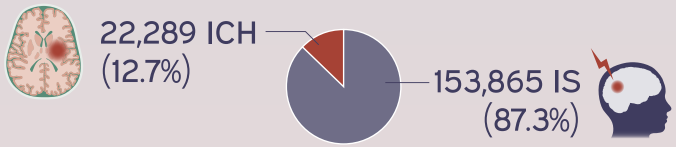

90-day functional outcome

Outcome	2010–12	2017–19	Adjusted trend
IS: mRS 0–2	49.2%	52.4%	OR 1.12 (1.09–1.16)
ICH: mRS 0–2	34.2%	34.3%	OR 0.96 (0.88–1.06)

90-day case fatality

Outcome	2010–12	2017–19	Adjusted trend
IS: CF 90d	13.8%	12.4%	HR 0.99 (0.95–1.02)
ICH: CF 90d	31.0%	30.4%	HR 1.00 (0.94–1.06)

Conclusion

Over 10 years, functional outcome after 90 days improved significantly in IS but not in ICH, while case-fatality remained unchanged.

Drescher, C., et al. European Stroke Journal, 2026 conrad.drescher@med.lu.se doi.org/xx.xxxx/xxxxxxxxxxxxxx
